# Effects of Polishing, Resin Coating, and Glaze Coating on the Surface Properties of a 3D‐Printed Splint Resin

**DOI:** 10.1111/jerd.70184

**Published:** 2026-05-05

**Authors:** İzim Türker Kader, Ecem Karacan, Berru Kursoğlu, Pınar Kursoğlu

**Affiliations:** ^1^ Department of Prosthodontics Bahçeşehir University, School of Dental Medicine Istanbul Turkey; ^2^ Department of Prosthodontics Yeditepe University, Faculty of Dentistry Istanbul Turkey; ^3^ Universität Witten/Herdecke, School of Dental Medicine Witten Germany

## Abstract

**Objective:**

The objective of this in vitro study was to evaluate the effects of different surface finishing protocols and post‐curing environments on the surface roughness (Ra) and Vickers hardness (VHN) of a 3‐dimensionally (3D)‐printed occlusal splint resin.

**Materials and Methods:**

Ninety‐six disc‐shaped specimens (Ø16 × 3 mm) were fabricated from a photopolymerizable splint resin using a stereolithography 3D printer. The specimens were assigned to four surface finishing groups: as‐printed (AP), polished (P), resin‐coated (RC), and glaze‐coated (GC). Each group was further subdivided according to the post‐curing environment, air (A) or glycerin (G), resulting in eight experimental groups (*n* = 12): AP‐A, AP‐G, P‐A, P‐G, RC‐A, RC‐G, GC‐A, and GC‐G. Post‐curing was performed at 60°C for 60 min. For the glycerin subgroups of the coated specimens (RC‐G and GC‐G), an initial curing period in air was applied before completing the post‐curing in glycerin to stabilize the applied coating layer. Ra was measured using a surface profilometer, and VHN was determined using a microhardness tester. The data were analyzed using two‐way ANOVA and post hoc Tukey HSD test (*α* = 0.05).

**Results:**

Surface finishing significantly affected Ra and VHN (*p* < 0.001), whereas the post‐curing environment had no effect (*p* > 0.05). AP‐A and AP‐G specimens showed the highest Ra, while GC‐A and GC‐G specimens exhibited the highest VHN (*p* < 0.001).

**Conclusions:**

Surface finishing is essential for optimizing surface properties of 3D‐printed occlusal splints. Although coating procedures, particularly glaze, were associated with higher surface hardness, polishing yielded smoother surfaces and provided consistent and clinically acceptable properties.

**Clinical Significance:**

Surface finishing plays an important role in the clinical performance of 3D‐printed occlusal splints. Although coating procedures yield high surface hardness, polishing provides smoother, more consistent surfaces, making it a practical and reliable option for routine clinical use.

## 1 Introduction

Occlusal splints are widely used to manage bruxism and temporomandibular disorders by maintaining neuromuscular balance and protecting teeth and prostheses from wear and fractures [1]. These appliances can be fabricated using conventional methods or digital manufacturing workflows [2].

Digital fabrication includes subtractive manufacturing, such as milling and additive manufacturing, also known as three‐dimensional (3D) printing [3, 4]. Among the available 3D‐printing technologies, stereolithography (SLA) has been widely used for the fabrication of occlusal splints because of its high accuracy and ability to produce detailed structures [5]. In SLA technology, an ultraviolet (UV) laser polymerizes liquid photopolymer resin layer by layer to form the desired object [6].

Although additive manufacturing enables efficient production of occlusal devices [7], its layer‐by‐layer fabrication process may compromise surface quality by creating characteristic step‐like textures that can increase roughness [8, 9, 10]. Surface roughness is an important parameter, as values exceeding 0.2 μm have been associated with increased microbial adhesion [11], while irregularities as small as 0.5 μm may be perceptible intraorally [12]. In addition to roughness, surface hardness is a critical property of occlusal splint materials because it reflects the material's resistance to deformation and surface damage under functional stresses [13, 14, 15].

Post‐processing is an essential step in 3D printing that affects the final properties of printed materials and typically includes rinsing, post‐curing and surface treatments such as polishing, coating, or glazing [6, 16]. Post‐curing can be performed in different environments, such as air or glycerin [17]. Oxygen present in the air may inhibit UV‐initiated free‐radical polymerization and result in partially cured surfaces; therefore, oxygen‐free conditions created using barriers such as glycerin have been suggested to enhance polymerization by limiting oxygen diffusion to the resin surface [18, 19, 20, 21].

Surface finishing procedures such as polishing, resin coating, and glaze coating have been proposed to improve the surface quality of 3D‐printed materials [22, 23, 24]. Previous studies have evaluated polishing and resin‐coating procedures for 3D‐printed occlusal device materials [5, 6, 7, 8, 17, 25], whereas glaze coatings have mainly been investigated in denture base resins [22, 26, 27, 28, 29]. However, the effects of different surface treatments and post‐curing environments on the surface properties of 3D‐printed splint resins remain unclear. Therefore, the present study aimed to evaluate the effects of different surface finishing protocols and post‐curing environments on the surface roughness and hardness of a 3D‐printed occlusal splint resin. The research hypotheses were as follows: (1) surface finishing procedures would result in differences in surface roughness and hardness, and (2) post‐curing environments would result in differences in surface roughness and hardness of the 3D‐printed splint resin.

## 2 Materials and Methods

**FIGURE 1:**
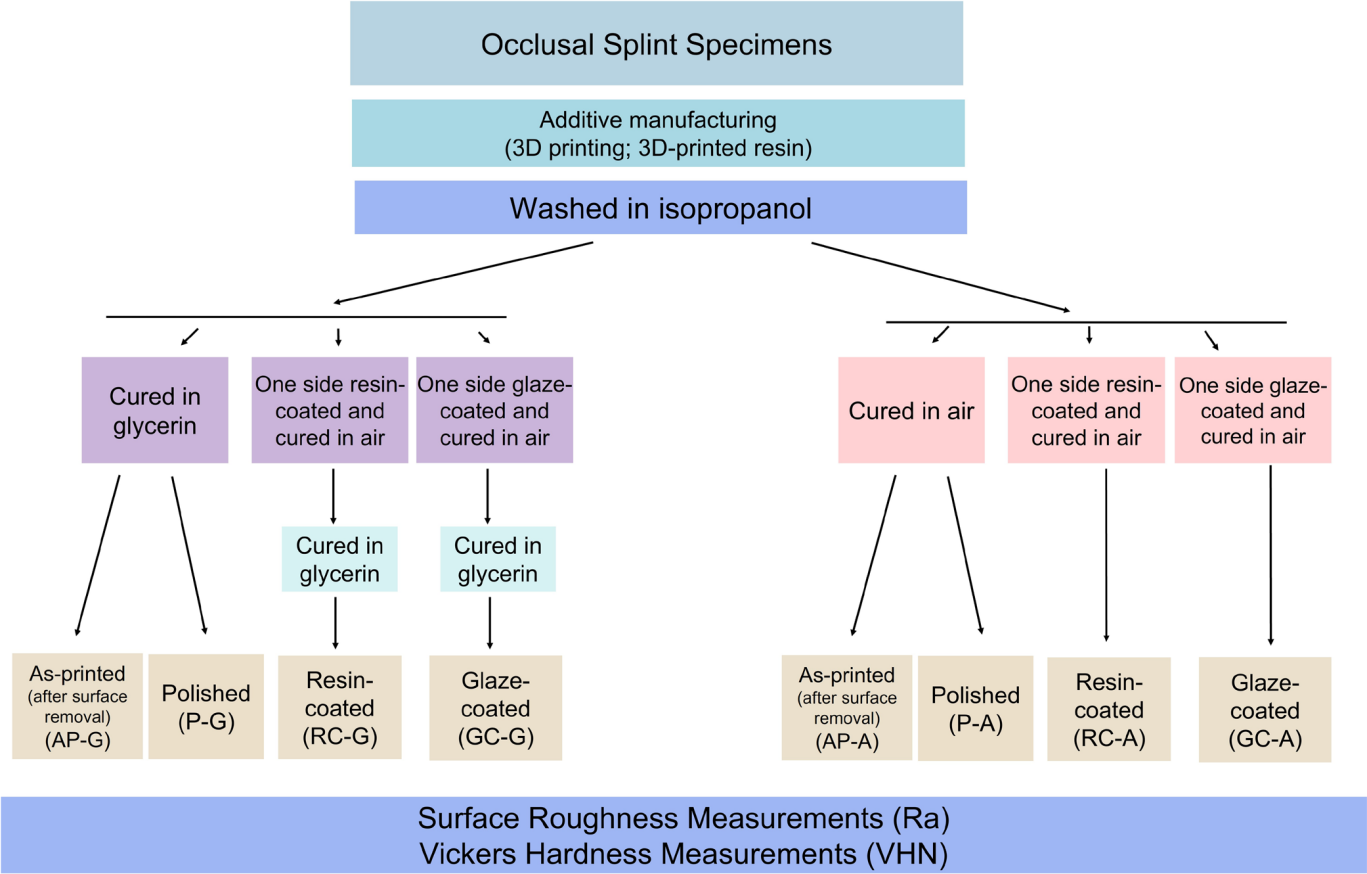
Study workflow. A: air; AP: as‐printed (after surface removal); G: glycerin; GC: glaze‐coated; P: polished; RC: resin‐coated.

The sample size per group (*n* = 10) was determined using a power analysis software program (G*Power v3.1.9.6; Heinrich Heine University Düsseldorf) with an effect size of *f* = 0.40, *α* = 0.05, and power = 0.90, based on a previous study [8]. Although the minimum required sample size was calculated as 10 specimens per group, 12 specimens were included in each group to ensure more reliable results and to strengthen the analysis. The study workflow is presented in Figure 1.

### 2.1 Specimen Preparation

A total of 96 disc‐shaped specimens (Ø16 × 3 mm) were designed using computer‐aided design (CAD) software (Preform Software; Formlabs) and exported as standard tessellation language (STL) files. The STL files were transferred to an SLA 3D printer (Form 3B+; Formlabs), and the specimens were printed using a photopolymerized acrylic occlusal splint resin (Dental LT Clear V2; Formlabs). All specimens were printed at a build angle of 45° and a layer thickness of 100 μm in accordance with the manufacturer's recommendations. This build angle was selected to standardize printing conditions and ensure consistency across experimental groups, and has been reported to provide a favorable balance between surface roughness and hardness [25, 30]. After printing, the specimens were washed in 99% isopropyl alcohol using a washing unit (Form Wash; Formlabs) to remove uncured resin. The printed specimens were then randomly divided into four surface finishing groups (*n* = 24): as‐printed, polished, resin‐coated, and glaze‐coated. Each group was further divided into two subgroups (*n* = 12) according to the post‐curing environment: air (A) or glycerin (G) resulting in 8 experimental groups. The groups were coded as AP‐A (as‐printed–air), AP‐G (as‐printed–glycerin), P‐A (polished–air), P‐G (polished–glycerin), RC‐A (resin‐coated–air), RC‐G (resin‐coated–glycerin), GC‐A (glaze‐coated–air), and GC‐G (glaze‐coated–glycerin).

### 2.2 Surface Finishing and Post‐Curing Procedures

Specimens in the as‐printed group (after superficial layer removal) served as the control group. After printing, the specimens were post‐cured either in air (AP‐A) or glycerin (AP‐G) at 60°C for 60 min using a curing unit (Form Cure; Formlabs). During preliminary testing, a soft, superficial layer was observed on the specimen surface, which interfered with reliable hardness measurements. Therefore, the surfaces were lightly abraded using P320 waterproof silicon carbide abrasive paper (English Abrasives & Chemicals Ltd.) with an automatic polishing machine (EcoMet 250; Buehler) operating at 400 rpm under water cooling to remove this superficial layer. This step was performed solely to remove the superficial layer and was not considered a surface finishing procedure. The abrasive papers were replaced after every 10 specimens to ensure consistent abrasion. No additional surface treatments were applied.

Specimens in the polishing group were post‐cured either in air (P‐A) or glycerin (P‐G) at 60°C for 60 min using the same curing unit. After post‐curing, the specimens were ground with P320 waterproof silicon carbide abrasive paper using the polishing machine (Ecomet 250) at 400 rpm under water cooling. Subsequently, the specimens were wet polished with a goat‐hair brush using a fine pumice slurry (Benco Dental) for 90 s at 1500 rpm. Final polishing was performed using a cotton disc and polishing paste (Ivoclar AG) for an additional 90 s.

For the resin‐coated group, a thin, uniform layer of the same printing resin was applied to one surface of each specimen before post‐curing. A brush was dipped once into a small container of resin and used to apply a thin coating to the specimen surface. For the RC‐A subgroup, the coated specimens were post‐cured in air at 60°C for 60 min using the curing unit. For the RC‐G subgroup, the coated specimens were initially cured in air for 12 min, followed by post‐curing in glycerin for 48 min at 60°C using the same curing unit. This two‐step protocol was used to prevent the flow of the applied resin layer, as observed during preliminary testing. After polymerization, excess uncured resin was gently wiped from the specimen surfaces.

For the glaze‐coated group, a thin layer of glaze material (Stain‐Glaze; ArmaDent) was applied to one surface of each specimen using the same brushing technique described for the resin‐coated group. For the GC‐A subgroup, the coated specimens were post‐cured in air at 60°C for 60 min using the curing unit. For the GC‐G subgroup, the coated specimens were initially cured in air for 12 min, followed by post‐curing in glycerin for 48 min at 60°C using the same curing unit. This protocol was implemented to stabilize the glaze layer and prevent material flow before curing in glycerin.

Following surface finishing and post‐curing procedures, all specimens were ultrasonically cleaned for 10 min in distilled water at room temperature using an ultrasonic cleaner (AS 8772 Ultrasonic Cleaner; General Home Orsay Company). After this procedure, the specimens were considered ready for further experimental evaluation.

### 2.3 Surface Roughness Measurements

Surface roughness (Ra) values were measured using a surface profilometer (Perthometer M1; Mahr) in accordance with the International Organization for Standardization (ISO) 21,920–2:2021 standard [31]. Five linear measurements were obtained from each specimen at 1‐mm intervals across a transverse length of 1.75 mm. The cut‐off value was set at 0.8 mm, and the stylus speed was 0.5 mm/s. The applied measurement force was approximately 0.7 mN. The mean Ra value for each specimen was calculated from the five measurements. Ra measurements were performed before the Vickers hardness test, and the indentation sites were selected in areas that did not overlap with the roughness measurement tracks.

### 2.4 Vickers Hardness Measurements

Surface hardness was evaluated using a Vickers hardness tester (Micromet 5114D; Buehler). A load of 0.98‐N was applied using a Vickers diamond indenter for 10 s [32]. Five indentations were made on each specimen in separate areas to avoid interference between measurements. The Vickers hardness number (VHN) was calculated using the following formula:

$$\mathrm{VHN}=1.854\left(F/D^{2}\right),$$

where *F* represents the applied load (*N*) and was converted to gram‐force for calculation, and *D* represents the diagonal length of the indentation (mm). The mean VHN value was calculated from the five measurements obtained from each specimen.

### 2.5 Statistical Analysis

Statistical analyses were performed using a statistical software program (IBM SPSS Statistics v25.0; IBM Corp). Mean values and standard deviations (SDs) were reported for each group. The Shapiro–Wilk test was used to assess the normality of the data, while the Levene test evaluated the homogeneity of variances. Two‐way ANOVA followed by Tukey HSD post hoc tests were used for inferential statistical analyses. Statistical significance was set at *α* = 0.05. All numerical data were expressed in accordance with standard significant figure rules, with mean values rounded to the same decimal precision as the corresponding SDs.

## 3 Results

Two‐way ANOVA revealed that surface finishing significantly affected both Ra and VHN (*p* < 0.001), whereas the post‐curing environment had no significant effect on either parameter (Ra: *p* = 0.545; VHN: *p* = 0.633). A significant interaction between surface finishing and post‐curing environment was observed for Ra (*p* = 0.014), but not for VHN (*p* = 0.092) (Table 1).

**TABLE 1:** Two‐way ANOVA results for the effects of surface finishing and post‐curing environment on surface roughness (Ra) and Vickers hardness (VHN).

Dependent variable	Factor	df	*F*	*p*	*η* ^2^
Ra	Surface finishing	3	39.82	**< 0.001**	0.576
Ra	Post‐curing environment	1	0.37	0.545	0.004
Ra	Surface finishing × post‐curing	3	3.76	**0.014**	0.114
VHN	Surface finishing	3	26.47	**< 0.001**	0.474
VHN	Post‐curing environment	1	0.23	0.633	0.003
VHN	Surface finishing × post‐curing	3	2.22	0.092	0.070

*Note:* All statistically significant *p*‐values are indicated in bold.

For Ra, since a significant interaction was detected, comparisons were performed within each surface finishing group (Table 2). The as‐printed group exhibited higher Ra values than all other groups, whereas polished, resin‐coated, and glaze‐coated groups demonstrated lower and comparable Ra values. Within each surface finishing group, no statistically significant differences were observed between air and glycerin post‐curing environments (*p* > 0.05).

**TABLE 2:** Comparison of post‐curing environments within each surface finishing group for surface roughness (Ra).

Surface finishing	Post‐curing environment		*p*
	Air (mean ± SD)	Glycerin (mean ± SD)	
As‐printed	0.20 ± 0.04^Aa^	0.14 ± 0.09^Ab^	**0.045**
Polished	0.04 ± 0.01^Ba^	0.05 ± 0.02^Ba^	0.325
Resin‐coated	0.06 ± 0.03^Ba^	0.08 ± 0.03^Ba^	0.316
Glaze‐coated	0.06 ± 0.04^Ba^	0.07 ± 0.04^Ba^	0.418
*p*	**< 0.001**	**< 0.001**	

*Note:* Different uppercase letters indicate significant differences among surface finishing groups within each column, and lowercase letters indicate differences between post‐curing environments within each row (*p* < 0.05). All statistically significant *p*‐values are indicated in bold.

**FIGURE 2:**
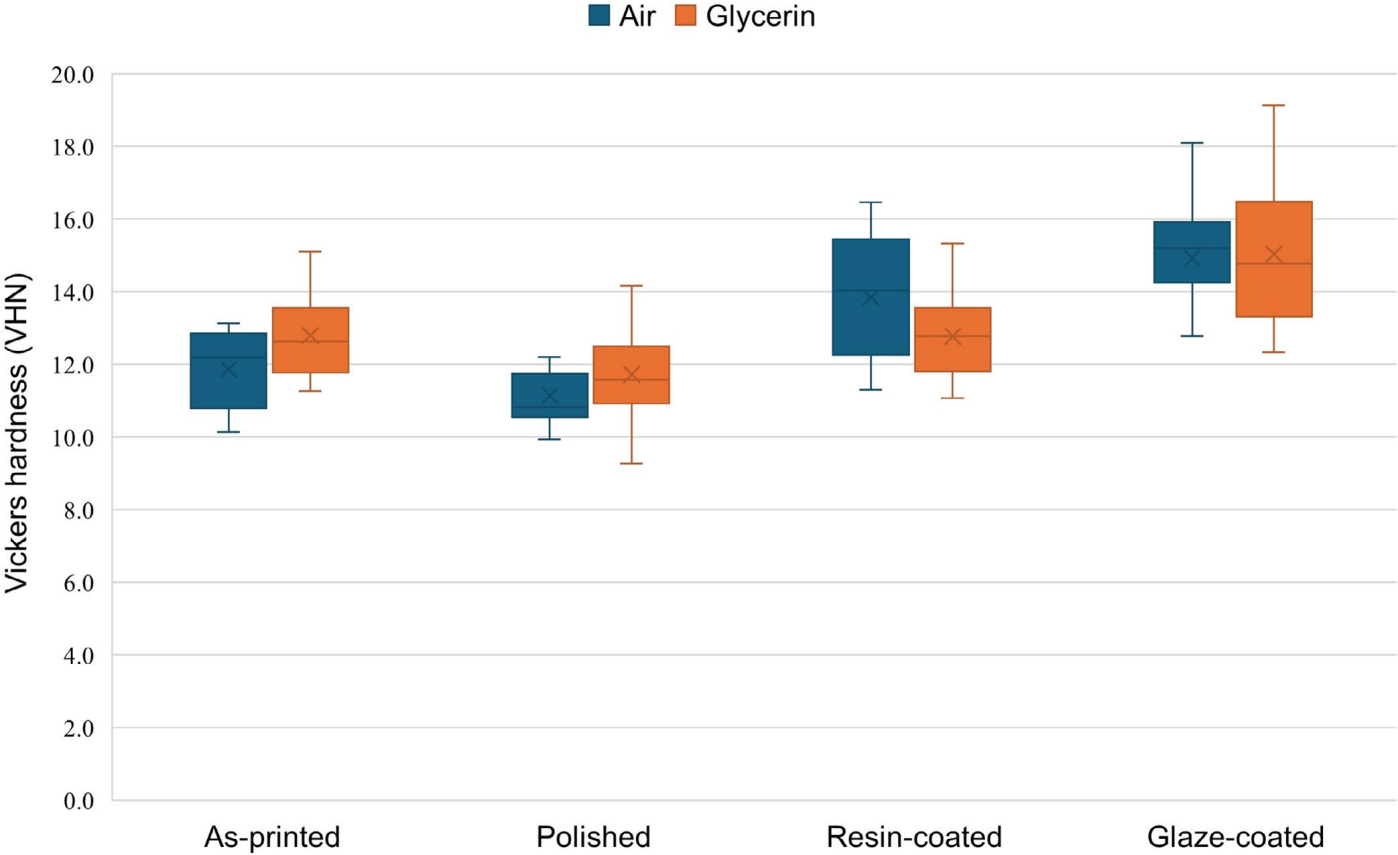
Boxplot of Vickers hardness (VHN) values according to surface finishing and post‐curing environment.

For VHN, as no significant interaction was found, the main effect of surface finishing was considered. Glaze‐coated specimens exhibited the highest values, whereas resin‐coated specimens showed intermediate values. Polished and as‐printed specimens generally demonstrated lower VHN values (Table 3). This pattern was consistent across both post‐curing environments (Figure 2).

**TABLE 3:** Descriptive statistics of Vickers hardness (VHN) according to surface finishing and post‐curing environment.

Group	Surface finishing	Post‐curing environment	VHN (mean ± SD)
AP‐A	As‐printed	Air	11.9 ± 1.1^a^
AP‐G	As‐printed	Glycerin	12.8 ± 1.2^ab^
P‐A	Polished	Air	11.1 ± 1.0^a^
P‐G	Polished	Glycerin	11.7 ± 1.3^a^
RC‐A	Resin‐coated	Air	13.9 ± 1.8^bc^
RC‐G	Resin‐coated	Glycerin	12.8 ± 1.2^ab^
GC‐A	Glaze‐coated	Air	14.9 ± 1.8^c^
GC‐G	Glaze‐coated	Glycerin	15.0 ± 1.9^c^

*Note:* Different superscript letters indicate statistically significant differences among groups (*p* < 0.05).

Abbreviations: A: air; AP: as‐printed (after surface removal); G: glycerin; GC: glaze‐coated; P: polished; RC: resin‐coated.

## 4 Discussion

The present study evaluated the effects of different surface finishing strategies and post‐curing environments on the surface roughness and hardness of a 3D‐printed occlusal splint resin. The findings supported the first research hypothesis, as surface finishing procedures resulted in differences in both surface roughness and hardness. As‐printed specimens exhibited higher roughness, whereas glaze‐coated specimens exhibited higher hardness. In contrast, the second research hypothesis was not supported, as the post‐curing environment did not result in differences in surface roughness and hardness.

The higher Ra values observed in as‐printed specimens are likely related to the inherent limitations of additive manufacturing [8, 9]. A previous study on 3D‐printed denture base resin reported that polishing and coating procedures can effectively reduce the characteristic staircase effect of layer‐by‐layer fabrication, resulting in smoother surfaces compared with untreated specimens [10]. In the present study, although all groups exhibited Ra values below the perceptibility threshold of 0.5 μm [12], the as‐printed specimens demonstrated values approaching the 0.2 μm threshold (AP‐A: 0.20 ± 0.04 μm) [11], indicating a borderline surface condition. In contrast, specimens subjected to polishing and coating procedures exhibited lower Ra values, remaining well below this threshold. These findings suggest that basic post‐curing alone may be insufficient to achieve clinically optimal surface smoothness in 3D‐printed splint materials.

Hardness is a critical parameter for occlusal splint materials, as these devices are continuously subjected to occlusal and parafunctional forces, reflecting resistance to surface deformation and functional loading [13, 14, 15]. In the present study, glaze‐coated specimens exhibited the highest VHN values, followed by resin‐coated groups. This may be attributed to the formation of a dense and highly cross‐linked superficial layer in coated specimens, which enhances resistance to indentation. Previous studies on 3D‐printed denture base resins have similarly reported increased hardness following glaze or resin coating applications [26, 28].

Surface finishing procedures influence surface characteristics of 3D‐printed materials through different mechanisms [16, 22, 23]. While polishing reduces surface irregularities via material removal, coating procedures smooth the surface by filling layer‐induced defects without substantial material loss [8, 19]. Although coating procedures may improve surface properties [8, 22, 24], their long‐term stability remains uncertain [27, 28, 29], as the applied coating layer may be susceptible to wear, delamination, and degradation under occlusal loading and routine cleaning procedures. These factors may compromise the durability of the coating over time and affect its long‐term clinical performance. Moreover, most of the available literature has focused on denture base materials [22, 26, 27, 28], and evidence regarding coating procedures in 3D‐printed occlusal splints remains limited.

Polishing does not require an additional layer and can be reapplied when necessary, making it a practical and repeatable approach in clinical settings. In addition, despite the lower hardness values observed, polishing provided comparable surface smoothness, suggesting a more balanced and clinically feasible approach for optimizing the surface properties of 3D‐printed occlusal splints. This finding suggests that polishing may be a reliable surface finishing approach for routine clinical applications.

Previous studies have suggested that post‐curing under oxygen‐inhibited conditions, such as glycerin, may enhance the surface and mechanical properties of resin‐based materials [8, 20, 21]. However, in the present study, no statistically significant differences were observed between air and glycerin conditions for either Ra or VHN. Although a significant interaction between surface finishing and post‐curing environment was observed for Ra, the overall effect of post‐curing environment on surface properties was limited. The lack of a significant effect of glycerin may be related to the reduced influence of the oxygen inhibition layer across all groups. Superficial layer removal in the as‐printed group and the surface finishing procedures applied in the other groups may have minimized this effect. In addition, the post‐curing temperature (60°C) may have contributed to sufficient polymerization, thereby limiting any additional effect of glycerin.

To strengthen the validity of this study, preliminary testing was conducted prior to the main experiments, and the methodology was refined accordingly. A soft superficial layer on the printed surfaces interfered with hardness measurements; therefore, a standardized surface preparation was implemented, as described in the Materials and Methods. This step was limited to superficial layer removal and was not considered a surface finishing procedure but rather a measure to ensure reliable hardness measurements. In addition, coating protocols were optimized through a two‐step curing procedure for resin‐ and glaze‐coated specimens to prevent material flow and achieve a more uniform surface layer. All specimens were fabricated using a 45° build angle to standardize printing conditions, ensure consistency across experimental groups, and comply with the manufacturer's recommendations. This orientation has been reported to provide a favorable balance between surface roughness and hardness [25, 30].

This in vitro study has several limitations. A single 3D printing system and one type of occlusal splint resin were evaluated. In addition, post‐curing conditions were limited to air and glycerin environments, and other post‐curing approaches were not investigated. Furthermore, surface finishing procedures were restricted to polishing and specific coating protocols. Future studies should explore different materials, post‐curing environments, and surface finishing techniques to further clarify their effects on the surface properties of 3D‐printed occlusal splint materials.

## 5 Conclusion

Within the limitations of this current study, it was concluded that:

1. Surface finishing procedures resulted in differences in surface roughness and hardness; polishing and coating procedures produced lower roughness values than as‐printed specimens, and glaze‐coated specimens showed the highest hardness values.
2. Post‐curing environment did not result in differences in surface roughness and hardness in the 3D‐printed splint resin.

## Funding

The authors have nothing to report.

## Disclosure

The authors do not have any financial interest in the companies whose materials are included in this article.

## Conflicts of Interest

The authors declare no conflicts of interest.

## Data Availability

The data that support the findings of this study are available from the corresponding author upon reasonable request.
